# Dr. Daniel Gaylord Mason

**Published:** 1912-09

**Authors:** 


Dr. Daniel Gaylord Mason, who had practiced in East Henrietta, Monroe Co., N. Y., for 28 years, died at his home, Aug. 13, aged 57. He was born in Walforth, Wayne Co., N. Y., July 9, 1855. He graduated in medicine, in 1879 at the New York University. Several years ago, he became infected during an operation and his health gradually declined, although he made a gallant fight for recovery and persisted in his medical duties till recently. He was a member of the State Society and the Monroe Co. branch, of the Central N. Y. Medical Association, and a charter member of the Rochester Academy of Medicine. He was formerly President of the Medical Society of the County of Monroe.

